# Microbial antigens-loaded myeloma cells enhance Th2 cell proliferation and myeloma clonogenicity via Th2–myeloma cell interaction

**DOI:** 10.1186/s12885-019-6469-4

**Published:** 2019-12-23

**Authors:** Faqing Tian, Bo Lu, Ziren Chen, Junru Liu, Delan Ji, Juheng Li, Meiqin Tang, Wei Zhu, Juan Li

**Affiliations:** 1 Department of Hematology, Longgang District People’s Hospital of Shenzhen, Shenzhen, 518172 Guangdong China; 2 Department of Hematology, The First Affiliated Hospital, Sun Yat-Sen University, Guangzhou, 510080 Guangdong China; 3 Department of Pathology, School of Basic Medicine, Guangdong Medical University, Dongguan, 523808 Guangdong China; 4 Department of Hematology, The Seventh Affiliated Hospital, Sun Yat-Sen University, Shenzhen, 518107 China; 5 Department of Hematological Oncology, Shenzhen University General Hospital, Shenzhen, 518055 China

**Keywords:** Myeloma, Microbial antigen, Antigen presenting cells, Th2 cell, Clonogenicity, MHC- II restriction

## Abstract

**Background:**

Myeloma cells retain B cell functions, considered to be potential antigen presenting cells, yet there is little information regarding promoting Th2 cell proliferation or the direct effects to myeloma on the Th2 cells stimulated by microbial antigens-loaded myeloma cells.

**Methods:**

Mixed lymphocyte reaction was used colorimetric assays via CCK8-kit. Surface molecular expression was performed by flow cytometry, cells sorting using microbeads. The concentrations of cytokines in serum were assessed using an ELISA kit. Clonogenic assay were performed in a methylcellulose culture system. Statistical analysis was assessed using the Student’s t-test or one-way analysis of variance for multiple comparisons test.

**Results:**

The expression of HLA-DR, CD80 and CD40 on RPMI8266 cell membrane surface was upregulated by interaction with interferon-γ and/or Bacillus Calmette-Guerin Vaccine (BCGV). RPMI8266 cells were able to induce the mixed lymphocyte reaction in a dose-dependent fashion. The Th2 ratio induced by RPMI8266 treated by BCGV and interferon-γ (treated-RPMI8266) cells was only slightly greater than by untreated-tumor cells, but the serum IL-4 level secreted by Th2 cells was markedly higher in treated-RPMI8266 cells group. Th2 cells stimulated by treated-myeloma cells could directly promote treated-myeloma cell clonogenicity in a dose-dependent manner. Anti-HLADR IgG2b completely blocked increased of IL-4 secretion by Th2 cells stimulated by treated-myeloma cells, while also blocked enhancing the clonogenicity of treated tumor cells stimulated by MM-Th2 cells.

**Conclusions:**

These results indicate that a novel mechanism of myeloma pathogenesis in myeloma cells could act as an APC to present microbial Ags to Th2 cells, promoting Th2 cell proliferation, consequently facilitating tumor development by close interaction between Th2 myeloma cells. Taken together, the microbial Ag presenting course of MM-Th2-MM interactions—restricted by MHC class-II—may result in tumor development such that all factors involved in the system could have a potential for myeloma therapeutic intervention.

## Background

Multiple myeloma (MM) is a B cell tumor that is characterized by the clonal expansion of malignant plasma cells in bone marrow [1]. A typical feature of MM is that it is clonally committed to the production and secretion of a specific immunoglobulin. Despite progress in therapeutic treatments for the disease, MM remains an incurable malignancy [2, 3]. Interactions involving stromal cells, as well as B- and plasma cell differentiation and growth factors—such as IL-6, BAFF and RANK—have been implicated in the development of MM [4–7]. Prior studies have demonstrated that heavy infiltration of human tumors by Dendritic cells (DCs), or polarization of Th2 cells in peripheral blood, is often correlated with an adverse prognosis [8–16]. DCs are able to directly enhance the clonogenicity of human MM by tumor–DC interactions [6]. Several studies have reported that Th2 cells have been confirmed to promote MM growth though myeloma cell-Th2 cell interactions [11, 17, 18]. Thus, antigen-presenting cells (APCs), for example DCs, and Th2 cells might play an important key in MM development.

Microbial antigens (Ags) have been demonstrated to be a potential cause of some tumors, and carcinogenesis may result from direct or indirect interaction between inflammatory cells/mediators with epithelial cells, stomal cells and extracellular matrix components, subsequently stimulating tumor angiogenesis [19–23]. Prior studies have confirmed that microbial Ags—such as Bacillus Calmette–Guerin Vaccine (BCGV) and tetanus toxoid antigen (TTA)—can enhance myeloma clonogenicity and are not just based on the level of humoral immunity [24–27]. Prior studies have also indicated that microbial Ags presented by DCs to Th2 cells (microbial Ags-Th2 cells) participate in MM pathogenesis after microbial Ags presentation by myeloma cells and DCs [17]. Therefore, the microbial Ags-Th2 cell transition is an important step in pathogenicity recurrence and MM development. Due to microbial antigens that are involved in many immune responses with affect a variety of immune cell functions, microbial Ags were posited to deeply affect myeloma biology from different aspects, possibly contributing to tumor cell growth.

Myeloma plasma cells, as APCs, can activate the immune response [17, 25]. These cells express a variety of surface markers, such as MHC-I, and surface antigens that are necessary for professional APCs, including adhesion and costimulation, but there are little expression of MHC-II, CD40 and CD80. Some studies have proven that MHC-II, CD40 and CD80 could be upregulated by interferon-γ (IFN-γ), tumor necrosis factor-α and microbial Ags, which is essential for APCs to activate Th2 cells [17, 24]. Myeloma cells are able to stimulate T cells and present the soluble antigens to autologous T cells, which are mainly Th1 cells that secrete IFN-γ [11, 25]. The ability of myeloma cells to stimulate and upregulate Th2 remains to be elucidated, similar to myeloma-stimilating-Th2 cells (MM-Th2) that act on myeloma cells to enhance clonogenicity. Th2 cells are polarized in MM patients and can induce MM cell proliferation [11, 17, 28, 29]. Therefore, the ability of myeloma cells to present microbial Ags to Th2 cells, and the role of these Th2 cells in promoting myeloma growth at same time, requires further investigation.

## Methods

### Healthy donor samples, patient samples and human myeloma cell lines

Peripheral blood and bone marrow aspirates were collected from healthy donors and four patients with MM. The RPMI8226 cell line was from the American Type Culture Collection (ATCC, Manassas, VA, USA) in 2015, and no further authentications were performed by the authors, not tested for mycoplasm contamination also.

### Mixed lymphocyte reaction (MLR)

We used the RPMI8226 multiple myeloma (MM) cell line as a model system to investigate whether MM cells are capable of presenting microbial antigens to T cells via MHC II, and whether that affects the response to T cells to MM cells in a mixed lymphocyte reaction (specific methods in a Additional file).

### Flow cytometry analysis

RPMI8266 cells were treated with IFN-γ and/or BCGV for 48 h. The myeloma cells were then stained with several defined antibodies and forward scatter flow cytometry (FCM) was used to measure the change in surface expression of CD80, CD86, CD40, CD54, and HLA-DR. After coculture with treated-MM cells for 5 days (MM:T = 1:1), CD4^+^CyIL-4^+^Th2 cells were analyzed by flow cytometry (FCM). Data were collected and analyzed using a FACScalibur flow cytometer and Cell Quest software (BD Biosciences, San Jose, CA, USA).

### ELISA assay

The concentrations of IL-4, IL-12 and IFN-γ in serum were assessed using an ELISA kit (R&D Systems, Minneapolis, MN, USA) according to the manufacturer’s instructions.

### Isolation of MM-Th2 cells

BCGV-loaded MM cells (treated-MM cells) were added to the T cells at a ratio of 1:1 in the presence of IL-2 (10 ng/ml; PeproTech, Rocky Hill, NJ, USA). After 5–7 days, MM-Th2 cells were isolated using CD4^+^ and CD294^+^(CRTH2) microbeads (Miltenyi Biotec, GmbH, Bergisch Gladbach, Germany) from the MM cell–T-cell coculture system according to the manufacturer’s instructions [30]. ^.^

### Clonogenic assays of RPMI8266 cells cocultured with Th2 cells

MM-Th2 cells (purity > 95%) irradiated with 30 Gy from a ^137^Cs source were added to treated-RPMI8266 cells at a ratio of 0:1, 1:1, 10:1, 20:1, 40:1 (tumor cells: 10,000/well), mixed completely and cocultured for 12 h for clonogenic assays. The clonogenic growth was evaluated by plating tumor cells in quadruplicate 35-mm^2^ tissue culture dishes, and for specific experimental methods, please refer to reference 17. Tumor colonies were counted after 2–3 weeks of culturing. The phenotype of the cells was confirmed by flow cytometry.

### Clonogenic assay of primary tumor cells

Mononuclear cells were isolated from the bone marrow samples of four MM patients using density gradient centrifugation, followed by treatment with IFN-γ and BCGV for 24 h. Then CD138^+^ and CD138^−^ fractions were isolated and plated with or without MM-Th2 cells at a ratio of 1:20 (MM:T) in a methylcellulose culture system as described in reference 17**.**

### MHC- II restriction

In order to study the role of MHC molecules in Th2 cell and treated-MM cell interactions, cells were incubated with mouse MoAb against HLA-DR (IgG2b; 1 μg/mL; Millipore, Billerica, MA, USA), mouse MoAb against MHC--I (IgG2b; 5 μg/mL; R&D Systems, Minneapolis, MN, USA), or control IgG2b (5 μg/mL) In order to assess the myeloma cell-T cell contact dependence of tumor cell clonogenicity, Transwell polyester membrane inserts (CLS3460, Corning, Inc., Corning, NY, USA) separating treated-RPMI8266 cells from MM-Th2 cells were compared with control inserts separating MM-Th2 cells from complete RPMI-1640 medium only.

### Statistical analysis

Differences between groups were assessed using the Student’s t-test or one-way analysis of variance for multiple comparisons test. The significance level was set at *P* < 0.05.

## Results

### Change in expression of surface markers on RPMI8266 cells

The expression of some markers (HLA-DR, CD40, CD80, CD86 and CD54) indicated that MM cells and Th2 cells are able to activate one another. On RPMI8266 cells, HLA-DR, CD40 and CD80 were expressed at a low level, while CD86 and CD54 were highly expressed. Treatment with IFN-γ for 48 h markedly increased the surface expression of HLA-DR, CD40 and CD80 (Additional file 1: Figure S1a) and BCGV alone upregulated HLA-DR, CD40 and CD80 expression (Additional file 1: Figure S1b). Therefore, IFN-γ and/ or exogenous Ag were able to directly alter the expression of surface molecules on RPMI8266 cells, suggesting that MM cells may act as APC in a similar manner to B cells.

### MLR of RPMI8266 cells

**Fig. 1:**
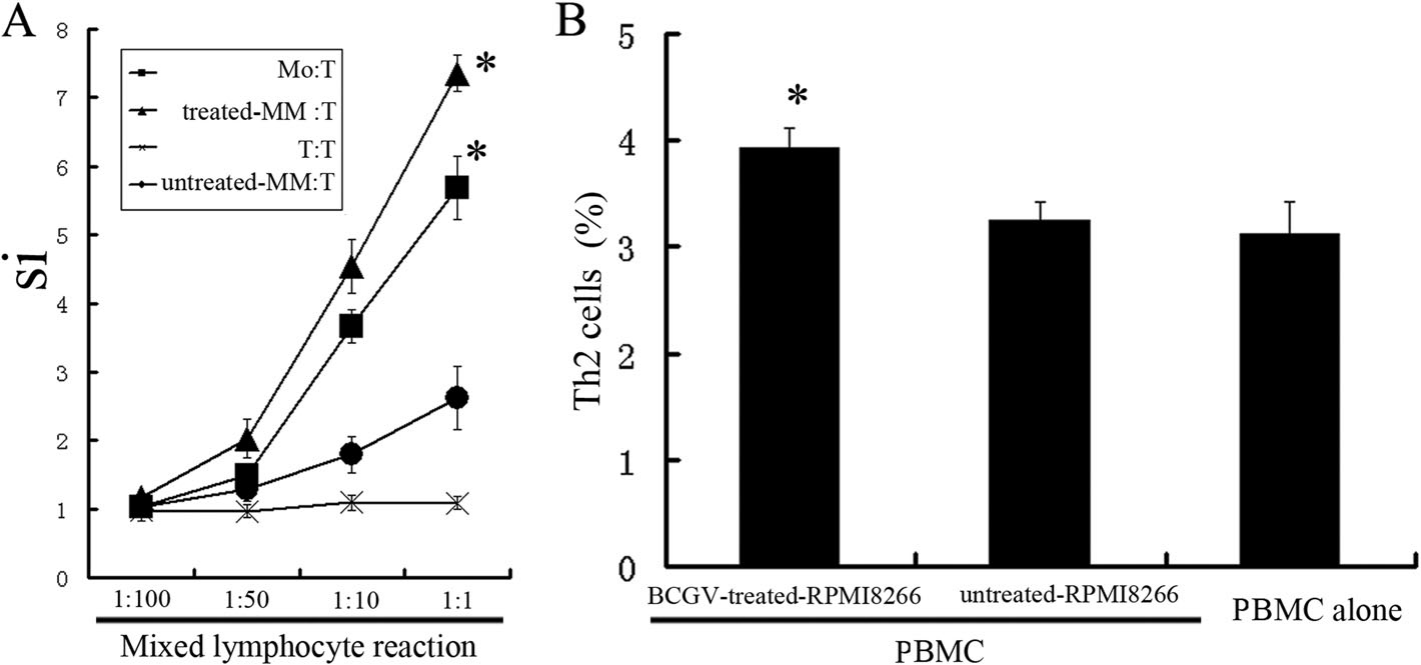
Mixed lymphocyte reaction and Th2 cell ratio. Colorimetric assays were performed to evaluate the MLR. SI (the stimulus index) = A _experimental well_/ A_control well_. (**A**) Treated-RPMI8266 cells (treated with IFN-γ and BCGV) induced proliferation of T cells in a dose-dependent manner, and treated-RPMI8266 cells had enhanced MLR in comparison to untreated RPMI8266 and T cells; * *p* < 0.05. (**B**,**C**) Th2 cells after MLR for 5 d (MM: T = 1:1) stained with CD4-FITC and IL-4-PE was analyzed by FCM. Bars represent the mean ± SEM (error bars) of four separate experiments. The ratio of Th2 cell was weakly increased, but the total number of T cell was significantly increased many times by treated-myeloma cells after MLR, so the absolute number of Th2 cell produced by treated-myeloma was more significantly increased. Th2 cell number calculation formula: *Absolute number*_*Th2*_ *= Total number*_*T cell*_*×Ratio*_*Th2*_

In order to assess the presentation of antigen to T cells by RPMI8266 cells, MLR was performed after RPMI8266 cells were treated with IFN-γ and BCGV for 48 h (treated-RPMI8266 cells). Treated-RPMI8266 cells induced proliferation of T cells in a dose-dependent manner. Treated-RPMI8266 cells had enhanced MLR than untreated RPMI8266 and T cells, and were similar to **monocyte cells (**Mo Cells) (Fig. 1a). FCM indicated that Th2 cell ratio produced by treated-RPMI8266 cells in PBMC was slightly higher than by untreated-RPMI8266 cells and by PBMC alone (Fig. 1b, Additional file 1: Figure S2).

### Cytokines levels

**Fig. 2:**
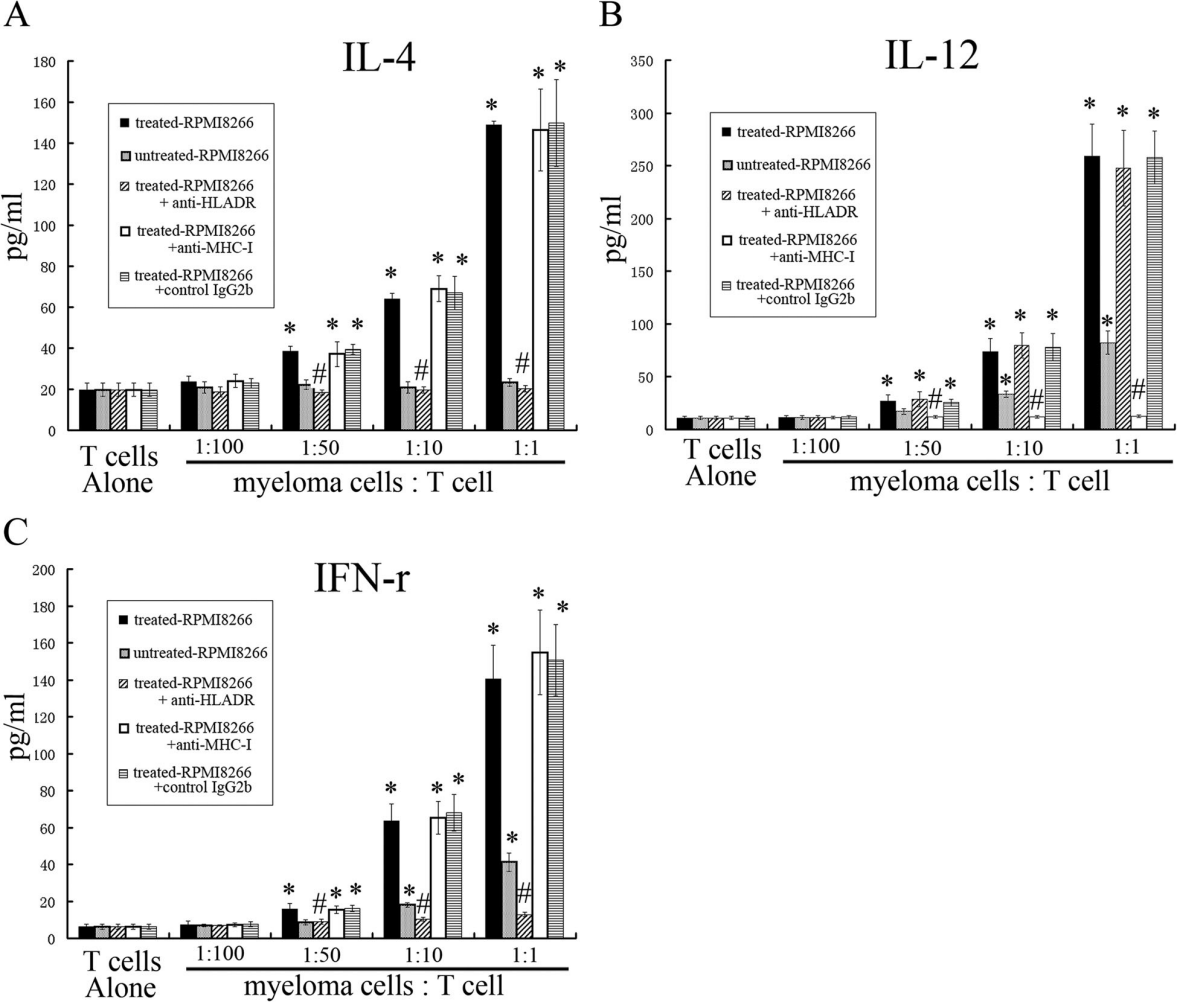
Cytokines levels determined by ELISA assay. IL-4, IFN- γ, and IL-12 are mainly secreted by Th2 cells, Th1 cells, and CD8 + T (CTL) cells, respectively, while Th2 cells cannot secrete IL-12 and IFN-γ, Th1 cells cannot secrete IL-12 and IL-4, and CTL cells cannot secrete IL-4 and IFN-γ. The levels of IL-4 and IFN-γ were not increased in the untreated-RPMI8266 group, but the levels did increase in treated-RPMI8266 cells group in a dose-dependent manner; * *p* < 0.05. (**A**,**C**) The increased IL-4 and IFN-γ levels were markedly repressed in the treated-RPMI8266 + anti-HLA-DR group but not in the anti-MHC-I and treated-RPMI8266 + control IgG2b groups; ^#^ *p* < 0.05. (**B**) The levels of IL-12 were significantly increased in every group except in the treated-RPMI8266 + anti-MHC-I group in a dose-dependent manner; * *p* < 0.05. The increasing of IL-12 level could be repressed in the treated-RPMI8266 + anti-MHC-I group but not in the treated-RPMI8266 + anti-HLA-DR and treated-RPMI8266 + control IgG2b groups; ^#^ *p* < 0.05

IL-4, IFN- γ, and IL-12 are mainly secreted by Th2 cells, Th1 cells, and CD8^+^T (CTL) cells, respectively, while Th2 cells cannot secrete IL-12 and IFN-γ, Th1 cells cannot secrete IL-12 and IL-4, and CTL cells cannot secrete IL-4 and IFN-γ. In order to further assess T cell proliferation, changes in MLR, IL-4, IL-12 and IFN-γ levels were determined by ELISA assay. IL-4 and IFN-γ levels were greatly increased in the treated-RPMI8266 cells group in a dose-dependent manner, while similar increases were not observed in other groups, except for the treated-RPMI8266 cells+anti-MHC-I and the treated-RPMI8266 cells+control IgG2b groups (Fig. 2a, c). IL-12 levels increased in all groups in a dose-dependent manner—except the treated-RPMI8266 cells+anti-MHC-I groups. IL-12 level was higher in untreated-RPMI8266 group than in the treated-RPMI8266 cells+anti-MHC-I group (Fig. 2b). These experiments demonstrated that myeloma cells promote the secretion of IFN-γ, IL-4 and IL-12 by activating Th1, Th2 and CTL cells, respectively. Since APC-presenting antigens promote the proliferation of CTL cells with MHC-I molecular restriction and the proliferation of Th1 and Th2 cells with MHC-II molecules. Therefore, anti-MHC-I can inhibit APCs from promoting CTL cell proliferation, and anti-MHC-II (anti-HLA-DR) can inhibit APCs from promoting Th1 and Th2 cell proliferation., which is characterized by decreased secretion of IFN-γ, L-12 and Il-4, respectively. Therefore, this experiment indirectly proves that myeloma cell is a kind of APC to produce mixed lymphocyte reaction, which can present BCG antigen to T cells, simultaneously greatly promoting the proliferation of Th1, CTL and Th2 cells and secretion of their corresponding cytokines, which restricted by MHC molecules.

### Stimulation of clonogenic growth of myeloma cells by Th2 cells

**Fig. 3:**
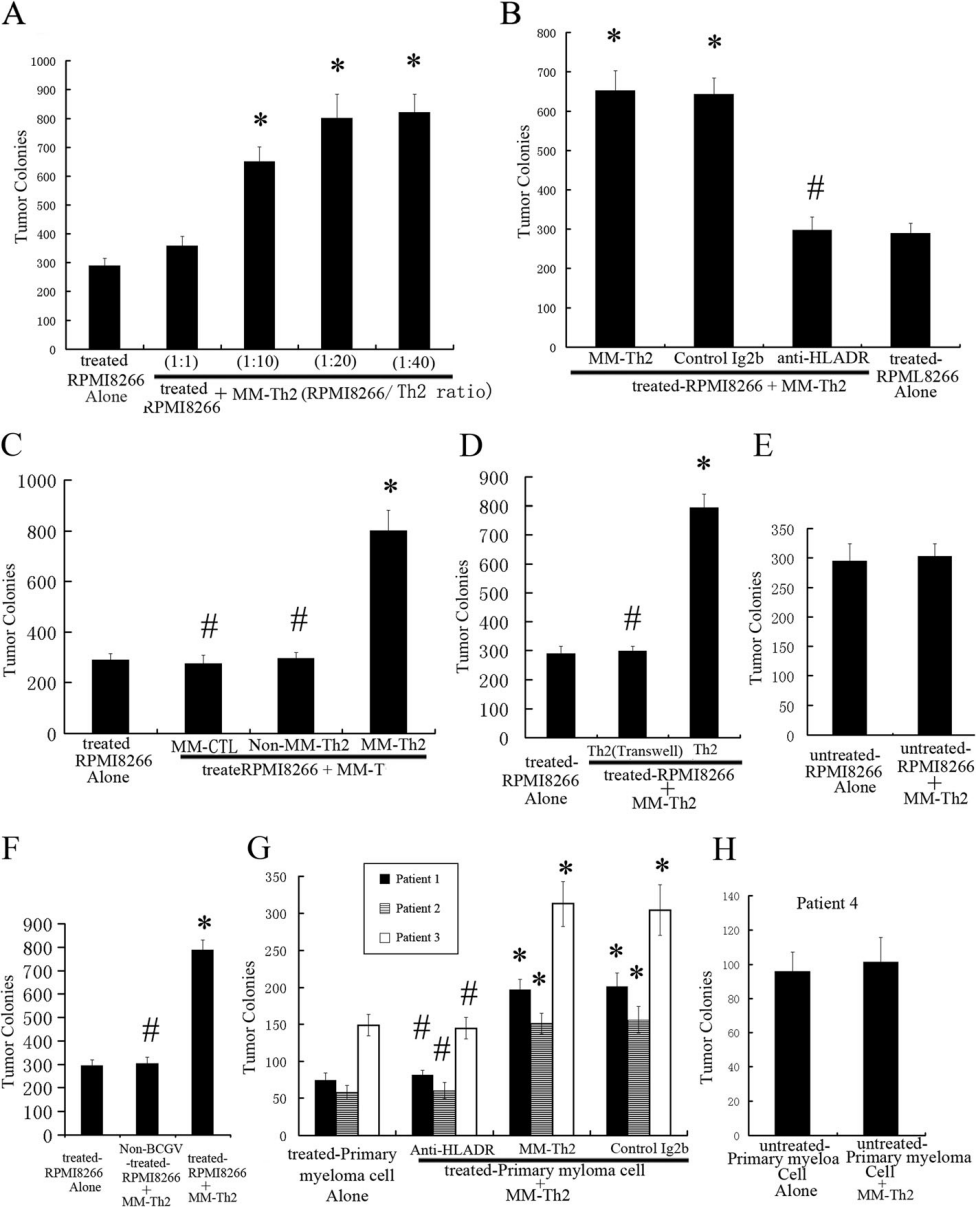
Human myeloma cell clonogenicity. Colonies were counted by microscope after 2–3 weeks of culturing. IFN-γ and BCGV-treated RPMI8266 cells were plated with or without MM-Th2 cells in a clonogenic assay (tumor cells: 10,000/well). Results are the mean ± SEM. (error bars) of three separate experiments; * *p* < 0.05. (**A**) MM-Th2 cells significantly increased the clonogenicity of treated-RPMI8266 cells in a Th2: tumor cell ratio-dependent manner; * *p* < 0.05. (**B**,**C**,**D**,**F**) MM-Th2 cells groups versus non-MM-Th2 cells groups (Th2/tumor ratio of 20:1). Clonogenicity was significantly enhanced by MM-Th2; * *p* < 0.05, but reduced by anti-HLA-DR and Transwell; ^#^ *p* < 0.05. (**C**) Clonogenicity of treated-RPMI8266 cells were not affected by MM-CTL cells and non-MM-Th2 cells compared with by MM-Th2; ^#^ *p* < 0.05. (**D**) Requirements for cell–cell contact. MM-Th2 cells were either plated along with treated-tumor cells or separated from treated-tumor cells by a transwell insert membrane. (**E**,**F**) Untreated-tumor cells and tumor cells treated only with IFN-γ (Non-BCGV-treated-PRMI8266) were not affected by MM-Th2 cells compared with treated-RPMI 8266; ^#^ *p* < 0.05. (**G**,**H**) Clonogenicity of primary myeloma cells (*n* = 4). Tumor cells treated with IFN-γ and BCGV (treated-primary myeloma cells) were cultured in the presence or absence of allogeneic Th2 cells (MM-Th2) at a ratio of 1:20 (tumor: Th2 cells, tumor cells: 100,000/well) in the clonogenic assay. MM-Th2 cells significantly increased the clonogenicity of treated-primary myeloma cells; * *p* < 0.05, but this effect was reduced by anti-HLA-DR; ^#^ *p* < 0.05. Untreated-primary myeloma cells were not affected by MM-Th2 cells. (b, g) Control IgG2b did not repress the enhancement of clonogenicity produced by MM-Th2 cells

In order to assess the response of RPMI8266 cells to Th2 cells, colorimetric assays were performed and suggested that MM-Th2 cells significantly increased the clonogenicity of treated-RPMI8266 cells in a Th2 cell:tumor cell ratio-dependent manner (Figs. 3a-d, f). In contrast, MM-Th2 cells had no impact on the clonogenicity of untreated-RPMI8266 and RPMI8266 cells treated with IFN-γ alone (Figs. 3 e-f, h), while non-MM-Th2 cells and MM-specific CTL cells (CD4^+^CD8^+^ T cells) had no impact on treated-RPMI8266 cells (Fig. 3c). MM-Th2 cell-mediated enhancement of tumor clonogenicity required close contact between tumor cells and Th2 cells, as the effect was not evident when the two cell populations were separated by a Transwell membrane (Fig. 3d). Therefore, interactions between treated-MM and MM-Th2 cells can directly promote MM clonogenicity. MM-Th2 cells were also able to increase the clonogenicity of primary myeloma cells treated by IFN-γ and BCGV (Figs. 3g).

### The effect of blocking MHC between Th2 cells and RPMI8266 cells

**Fig. 4:**
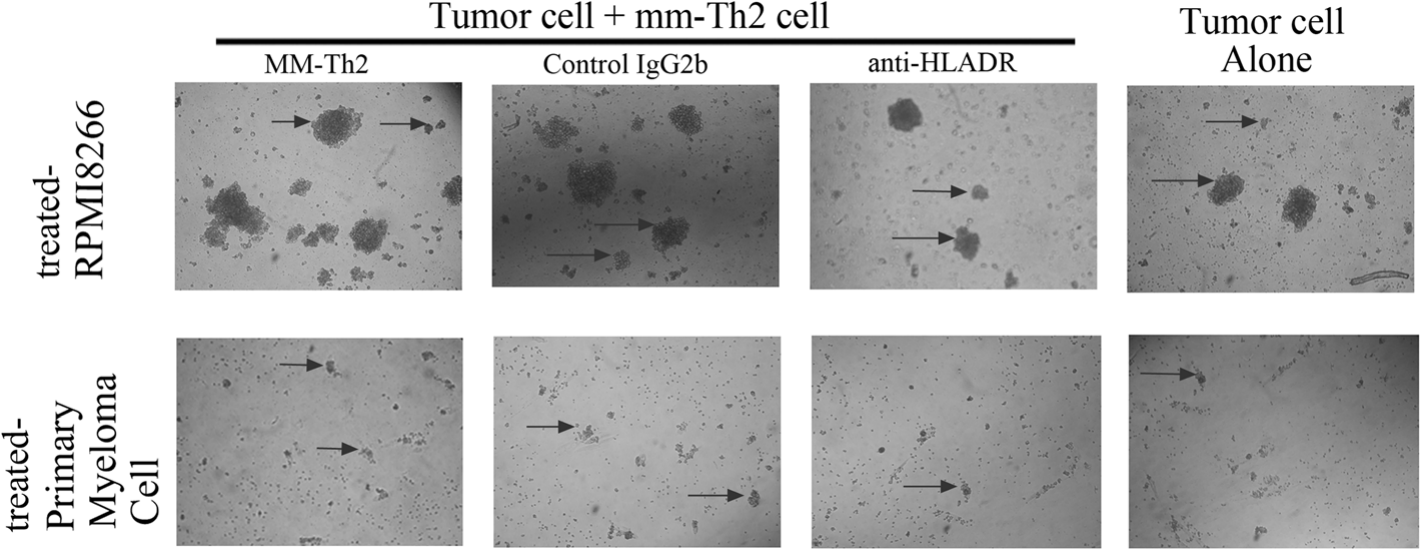
Tumor colony appearance. The clonogenicity assays were performed in methylcellulose. Treated tumor cells were plated with MM-Th2 cells (Tumor + Th2), without MM-Th2 cells (tumor Alone), with MM-Th2 cells and anti-HLA-DR (Anti-HLA-DR), or with MM-Th2 cells and control IgG2b (control IgG2b). Micrographs represent the appearance of colonies at low power. (Upper panel) Appearance of colonies of treated-RPMI8266 cells (tumor cells: 10,000/well). (Lower panel) Appearance of colonies of primary myeloma cells from bone marrow (tumor cells: 100,000/well). Arrow: pointing to tumor colony

Prior studies have confirmed that myeloma cells can present Ag peptides to T cells, which are MHC restricted, and myeloma colony growth that is promoted by Th2 cells is mainly restricted to MHC class-II. In order to gain insight into the role of MHC-II in the interaction between MM-Th2 cells and treated-RPMI8266 cells, MoAbs against MHC-II (anti-HLA-DR MoAb) were used to inhibit Th2 cell proliferation induced by treated-RPMI8266 cells, as well as the enhancement of MM-Th2 cell-stimulated clonogenicity of treated-RPMI8266 cells. IL-12 levels were decreased by MoAbs against MHC-I, suggesting that CD8^+^T cell proliferation induced by treated-RPMI8266 cells could be inhibited by MoAbs against MHC-I (Figs. 2b). Furthermore, pretreatment with an anti-HLA-DR MoAb could abolish the enhancement in tumor clonogenicity caused by MM-Th2 cells (Figs. 3, 3, 4b g). Therefore, cell proliferation induced by Th2 cell–myeloma cell interactions was primarily restricted to MHC class-II.

## Discussion

Several studies have suggested that a proportion of malignant plasma cells retain B cell functions, may possess stem cell characteristics, and must interact with Th2 cells to form colonies [11, 17, 25]. In our assays, although Th2 cell ratio promoted by treated-myeloma cells was only slightly higher by FCM, the total number of T cell was significantly increased many times by treated-myeloma cells after MLR, so the absolute number of Th2 cell promoted by treated-myeloma was more significantly increased. Th2 cell absolute number calculation formula: *Absolute number*_*Th2*_ *= Total number*_*T cell*_*×Ratio*_*Th2*_. IL-4 is secreted only by Th2 cells, and its level secreted was markedly higher in treated-RPMI8266 cells group, also suggesting that proliferation of Th2 cells was greatly promoted by treated-RPMI8266 cells. Therefore, malignant plasma cells could induce MLR, promoting CD8^+^T (CTL) and Th1 cell proliferation and could also promote Th2 cell proliferation, suggesting that myeloma cells present Ags to T cells, including Th2 cells. In short, myeloma cells may be regarded as APC, similar to B cell s, which is consistent with previous reports [11, 17, 25]. However, the MLR of untreated-MM cells was greatly reduced in comparison to treated-MM, and untreated-MM cells did not increase IL-4 levels secreted by Th2 cells, but it did in treated-MM cells. This may suggest that myeloma cells did not directly promote Th2 cell proliferation or induce naïve T cells to Th2 cells until myeloma cells were treated with IFN-γ and BCGV. Prior studies have confirmed that U266 and primary myeloma cells express B cell membrane markers, including high levels of CD86 and CD54 and detectable levels of HLA-DR, CD40 and CD80, which can be upregulated by IFN-γ combined with microbial Ags or by BCGV alone [17, 25]. In these assays, the expression of HLA-DR, CD40 and CD80 of RPMI8266 cells was also increased by IFN-γ and BCGV or by BCGV alone, which is essential for APCs to activate Th2 cells, so, the treated-RPMI8266 like B cells could cause Th2 cell proliferation.

Th2 cells are polarized in patients with MM and can cause MM cell proliferation or clonogenicity [11, 17, 28, 29]. DCs present microbial Ags to Th2 cells, which then directly promotes myeloma clonogenicity when myeloma encounter the same Ags by Th2 cell-myeloma cell direct interaction [16]. In these assays, myeloma cells acted as an APC that directly presents microbial Ags to promote Th2 cell proliferation (MM-Th2 cells), which in turn directly promoted myeloma clonogenicity. In MM, DCs present microbial Ags to Th2 cells which are involved in pathogenecity, recurrence and development of MM, while myeloma cells can present microbial Ags to Th2 cells, subsequently stimulating tumor clonogenicity. However, these results do not exclude other mechanisms for MM pathogenesis. Blocking T-cell receptor-MHC-II interaction by anti-MHC-II MoAb (anti-HLADR) completely repressed Th2 cell IL-4 secretion that was promoted by treated-myeloma cells. This decreases the amount of tumor clonogenicity stimulated by MM-Th2 cells, indicating that MHC-II restriction could be mediated by Th2 cell and treated-MM cell interactions. This data is similar to previous assays, which detailed that the interaction between Th2-myloma cells is similar to interaction between Th2 cell and B cell. These interactions require T-cell receptor-MHC-II and CD40-CD40L binding, as well as other molecule interaction such as adhesion molecules (CD54-CD11a interaction), costimulatory molecules (B7-CD28 interaction) or Toll-like receptors [17, 31–33]. The transwell assays further confirmed that the stimulation of clonogenic growth of myeloma cells by Th2 cells required close contact between tumor cells and Th2 cells, as this effect was not evident when the two cell populations were separated by a Transwell membrane. Therefore, Th2 and MM cells require close contact just like Th2 cell and B cell interactions, which are MHC class-II restricted.

Similar to previous assays [17], microbial Ags were confirmed to play an important role in the development of myeloma, but antigens presented by primary MM cells are likely to be different from what is being presented after BCGV treatment. BCGV treatment is likely to be not only a source of peptide antigen, but to stimulate MM cells through TLRs in vivo and vitro. In our assays, microbial Ags not only directly increased the expression of MHC-II, CD40 and CD80 and changing tumor biology, but the direct presentation of microbial Ags by myeloma cells to Th2 cells could directly stimulate myeloma clonogenicity, much different from the conclusions in prior studies [19–21]. To our knowledge, this is the first observation that microbial Ags could directly affect myeloma cells to promote tumor development, as myeloma cells could present the Ags to Th2 cells. During this course, microbial Ags to promote the expression of MHC-II, CD40 and CD80 in myeloma cells may be the most critical step to stimulate tumor clonogenicity. In short, microbial antigens-loaded myeloma cells could enhance Th2 cell proliferation and myeloma clonogenicity via Th2–myeloma cell interaction.

## Conclusions

In conclusion, this study presents evidence for a novel mechanism of MM pathogenesis, whereby myeloma cells act as APS to present microbial Ags to Th2 cells, in turn facilitating tumor development by Th2 cells-myeloma cells via close interactions. Thus, the microbial Ag presenting course of MM-Th2-MM interactions—restricted by MHC class-II—may result in tumor development such that every factor involved in the system may be potential targets for new myeloma therapies and interventions.

## Supplementary information

**Additional file 1:** Mixed lymphocyte reaction (MLR). Peripheral blood monocytes (PBMC) were isolated from freshly-collected heparin-treated blood by density gradient centrifugation. Subsequently, 5 × 10^6^ cells/mL were cultured for 2–4 h to permit cell adherence and the adherent cells acted as monocytes. Nonadherent cells were further depleted of normal B cells using CD19 micro beads (Miltenyi Biotec), which acted as T cells. T cells in the monocyte-depleted and B-cell-depleted population were > 95%. CD14^+^ monocytes in adherent cells were > 60%. RPMI8266 cells treated with IFN-γ (Shanghai Prime Gene Bio-Tech Co., Shanghai, China) and BCGV for 48 h (treated-RPMI8266) were irradiated with 30 Gy from a ^137^Cs source and added in a gradient as stimulators to a 96-well round-bottomed microtitre plate, each well containing 200 μL of 1× 10^5^ allogenic T cells (experimental well). The well of allogenic T cells alone was as control. Colorimetric assays were performed in order to evaluate the proliferation of T cells by treated-MM cells on day 5. Next, 20 μL of CCK-8 (Cell Counting Kit-8; Dojindo, Kumamoto, Japan) were added to each well, followed by incubation at 37 °C for 3–4 h in a humidified CO_2_ incubator for quantitative analysis of cell viability. The absorbance (A) at 450 nm wavelength (630 nm as control of 450 nm) was monitored with a microplate reader (ELX800, BIO-TEK, USA), then to calculate the stimulus index (SI). *SI = A*_*experimental well*_*/ A*_*control well*_. **Figure S1.** Surface Ags on RPMI8266 cells treated by IFN-γ or BCGV**.** Surface HLA-DR, CD40, CD80, CD86 and CD54 expression on tumor cells were examined by flow cytometry. (a) Treatment of RPMI8266 cells with IFN-γ for 48 h markedly increased HLA-DR, CD40 and CD80 expression, but CD40 and CD80 expressions were at a low level. RPMI8266 cells expressed high levels of CD86 and CD54. (b) Expression of HLA-DR, CD40 and CD80 was increased after treatment of RPMI8266 cells with BCGV; * *p* < 0.05.**Figure S2.** Mixed lymphocyte reaction and Th2 cell ratio**.** Th2 cells after MLR for 5 d (MM: T = 1:1) stained with CD4-FITC and IL-4-PE was analyzed by FCM. The ratio of Th2 cell was weakly increased

## Data Availability

All data generated or analyzed during this study are included in this published article.

## Abbreviations

APC: Antigen-presenting cell; antigen: Ag
BCGV: Bacillus Calmette–Guerin Vaccine
CCK8: Cell Counting Kit-8
DC: Dendritic cells
FCM: Flow cytometry analysis
IFN-γ: Interferon-γ
MLR: Mixed lymphocyte reaction
MM: Multiple
MM-Th2: Myeloma-stimilating-Th2 cells
PBMC: Peripheral blood monocyte
SI: Stimulus index
TTA: Tetanus toxoid antigen
