# Supplementary material for: Microbial antigens-loaded myeloma cells enhance Th2 cell proliferation and myeloma clonogenicity via Th2–myeloma cell interaction
Source: BMC Cancer. 2019 Dec 23;19:1246. doi: 10.1186/s12885-019-6469-4 (PMC6929311; doi:10.1186/s12885-019-6469-4)
Supplement: Supplementary file 1 — Additional file 1: Mixed lymphocyte reaction (MLR). Peripheral blood monocytes (PBMC) were isolated from freshly-collected heparin-treated blood by density gradient centrifugation. Subsequently, 5 × 10^6^ cells/mL were cultured for 2–4 h to permit cell adherence and the adherent cells acted as monocytes. Nonadherent cells were further depleted of normal B cells using CD19 micro beads (Miltenyi Biotec), which acted as T cells. T cells in the monocyte-depleted and B-cell-depleted population were > 95%. CD14+ monocytes in adherent cells were > 60%. RPMI8266 cells treated with IFN-γ (Shanghai Prime Gene Bio-Tech Co., Shanghai, China) and BCGV for 48 h (treated-RPMI8266) were irradiated with 30 Gy from a 137Cs source and added in a gradient as stimulators to a 96-well round-bottomed microtitre plate, each well containing 200 μL of 1× 10^5^ allogenic T cells (experimental well). The well of allogenic T cells alone was as control. Colorimetric assays were performed in order to evaluate the proliferation of T cells by treated-MM cells on day 5. Next, 20 μL of CCK-8 (Cell Counting Kit-8; Dojindo, Kumamoto, Japan) were added to each well, followed by incubation at 37 °C for 3–4 h in a humidified CO2 incubator for quantitative analysis of cell viability. The absorbance (A) at 450 nm wavelength (630 nm as control of 450 nm) was monitored with a microplate reader (ELX800, BIO-TEK, USA), then to calculate the stimulus index (SI). SI = A experimental well/ Acontrol well. Figure S1. Surface Ags on RPMI8266 cells treated by IFN-γ or BCGV. Surface HLA-DR, CD40, CD80, CD86 and CD54 expression on tumor cells were examined by flow cytometry. (a) Treatment of RPMI8266 cells with IFN-γ for 48 h markedly increased HLA-DR, CD40 and CD80 expression, but CD40 and CD80 expressions were at a low level. RPMI8266 cells expressed high levels of CD86 and CD54. (b) Expression of HLA-DR, CD40 and CD80 was increased after treatment of RPMI8266 cells with BCGV; * p < 0.05.Figure S2. Mixed lymphocyte reacti [file 12885_2019_6469_MOESM1_ESM.docx]

Addttional file:

**Mixed lymphocyte reaction (MLR)**

Peripheral blood monocytes (PBMC) were isolated from freshly-collected heparin-treated blood by density gradient centrifugation. Subsequently, 5 × 10^6^ cells/mL were cultured for 2–4 h to permit cell adherence and the adherent cells acted as monocytes. Nonadherent cells were further depleted of normal B cells using CD19 micro beads (Miltenyi Biotec), which acted as T cells. T cells in the monocyte-depleted and B-cell-depleted population were >95%. CD14^+^ monocytes in adherent cells were >60%. RPMI8266 cells treated with IFN-γ (Shanghai Prime Gene Bio-Tech Co., Shanghai, China) and BCGV for 48 h (treated-RPMI8266) were irradiated with 30 Gy from a ^137^Cs source and added in a gradient as stimulators to a 96-well round-bottomed microtitre plate, each well containing 200 µL of 1× 10^5^ allogenic T cells (experimental well). The well of allogenic T cells alone was as control. Colorimetric assays were performed in order to evaluate the proliferation of T cells by treated-MM cells on day 5. Next, 20 μL of CCK-8 (Cell Counting Kit-8; Dojindo, Kumamoto, Japan) were added to each well, followed by incubation at 37 °C for 3-4 h in a humidified CO_2_ incubator for quantitative analysis of cell viability. The absorbance (A) at 450nm wavelength (630 nm as control of 450nm) was monitored with a microplate reader (ELX800, BIO-TEK, USA), then to calculate the stimulus index (SI). *SI = A _experimental well_/ A_control well_*.

Figure **Legends**

**Figure S1. Surface Ags on RPMI8266 cells treated by IFN-γ or BCGV.** Surface HLA-DR, CD40, CD80, CD86 and CD54 expression on tumor cells were examined by flow cytometry. (a) Treatment of RPMI8266 cells with IFN-γ for 48 h markedly increased HLA-DR, CD40 and CD80 expression, but CD40 and CD80 expressions were at a low level. RPMI8266 cells expressed high levels of CD86 and CD54. (b) Expression of HLA-DR, CD40 and CD80 was increased after treatment of RPMI8266 cells with BCGV; * *p* < 0.05.

**Figure S2. Mixed lymphocyte reaction and Th2 cell ratio.** Th2 cells after MLR for 5 d (MM: T = 1:1) stained with CD4-FITC and IL-4-PE was analyzed by FCM. The ratio of Th2 cell was weakly increased.
